# Constraint-based modelling predicts metabolic signatures of low and high-grade serous ovarian cancer

**DOI:** 10.1038/s41540-024-00418-5

**Published:** 2024-08-24

**Authors:** Kate E. Meeson, Jean-Marc Schwartz

**Affiliations:** School of Biological Sciences, University of Manchester, Manchester, UK

**Keywords:** Cancer, Computational biology and bioinformatics, Computer modelling

## Abstract

Ovarian cancer is an aggressive, heterogeneous disease, burdened with late diagnosis and resistance to chemotherapy. Clinical features of ovarian cancer could be explained by investigating its metabolism, and how the regulation of specific pathways links to individual phenotypes. Ovarian cancer is of particular interest for metabolic research due to its heterogeneous nature, with five distinct subtypes having been identified, each of which may display a unique metabolic signature. To elucidate metabolic differences, constraint-based modelling (CBM) represents a powerful technology, inviting the integration of ‘omics’ data, such as transcriptomics. However, many CBM methods have not prioritised accurate growth rate predictions, and there are very few ovarian cancer genome-scale studies. Here, a novel method for CBM has been developed, employing the genome-scale model Human1 and flux balance analysis, enabling the integration of in vitro growth rates, transcriptomics data and media conditions to predict the metabolic behaviour of cells. Using low- and high-grade ovarian cancer, subtype-specific metabolic differences have been predicted, which have been supported by publicly available CRISPR-Cas9 data from the Cancer Cell Line Encyclopaedia and an extensive literature review. Metabolic drivers of aggressive, invasive phenotypes, as well as pathways responsible for increased chemoresistance in low-grade cell lines have been suggested. Experimental gene dependency data has been used to validate areas of the pentose phosphate pathway as essential for low-grade cellular growth, highlighting potential vulnerabilities for this ovarian cancer subtype.

## Introduction

Ovarian cancer (OC) is the seventh most common cancer in women, accompanied by a staggering 1 in 75 lifetime risk^1^^,^^2^. The disease is typically diagnosed at late-stage, where less than 1 in 3 women will survive 5 years, thus OC signifies a major clinical burden^2^. Although it may also develop from sex-cord stromal or germ cells, over 90% of OCs are epithelial in origin^2^. Epithelial OC itself is an extremely heterogeneous disease, which may be split into five main subtypes: high-grade serous (HGSOC), endometrioid, clear cell, mucinous and low-grade serous (LGSOC)^3^. HGSOC and LGSOC represent subtypes with contrasting phenotypes and genomic profiles. HGSOC—the more aggressive subtype—makes up around 70% of cases, with ubiquitous TP53 mutations, homologous recombination deficiency in 50% of cases and BRCA1/2 inactivation^2–4^. On the other hand, LGSOC constitutes less than 5% of ovarian tumours, and is defined by BRAF, KRAS and PTEN mutations^2,3^.

Standard, first-line treatment for OC involves cytoreductive, debulking surgery, accompanied by platinum-based chemotherapy, such as cisplatin, with paclitaxel^5^. The heterogeneity of OC complicates treatment strategies. Patients with high-grade tumours demonstrate a more positive initial response, however unfortunately relapse is common^3^. Tumours can be subtyped according to histology and pathogenesis (Vang et al.^6^), however, if metabolic characterisation was also considered, this could permit a more detailed prognosis, given metabolism is directly linked to therapeutic response and the mechanisms of cancer progression.

Metabolism dictates a broad scope of tumour behaviour, including but not limited to nutrient demand, energy production, cellular growth and drug response. To understand tumour metabolism, we can take direct measurements of the flux through metabolic pathways, as well as make predictions based on the expression of metabolic enzymes. Both direct flux measurement and enzyme expression are two inputs which can be used to tailor genome-scale models (GEMs). Metabolic features of HGSOC vs LGSOC have been reviewed in recent years, revealing distinguishing features of carbon and energy metabolism, as well as fatty acid metabolism. For example, glycolysis is a central focus in cancer metabolism, and although the Warburg effect (preference for aerobic glycolysis even in the presence of oxygen) has been recognised as a Hallmark of cancer, it has been suggested that in some subtypes, oxidative phosphorylation is the preferred pathway^7^^,^^8^. Furthermore, the rate of flux through glycolysis may differ between HGSOC and LGSOC, and high-grade tumours might upregulate oxidative phosphorylation to a greater extent^8^. Aside from carbon metabolism, numerous enzymes involved in fatty acid metabolism have been identified as key drivers, for example, the overexpression of fatty acid synthase (FASN) has been associated with poor prognosis in OC, as well as other enzymes such as ATP-citrate lyase and acetyl-CoA carboxylase (ACC)^9^.

Omics data collection has become widely used to characterise dysregulated pathways in diseases, and GEMs are efficient for the translation of gene and protein expression data onto an in silico model. In principle, GEMs can be thought of as a multi-dimensional network, containing all of the known metabolic reactions and associated enzymes of a particular organism, serving as platforms onto which gene and protein expression data can be integrated to construct a context-specific, personalised GEM. The process of producing these enzyme-constrained GEMs (ecGEMs) is called constraint-based modelling (CBM), and such ecGEMs can be manipulated via genetic engineering simulations, such as gene overexpression or deletion, reaction inhibition, essentiality predictions and drug simulations^10^. Thereby, ecGEMs enable us to make predictions to be tested with experimental work, which can save time and resources, potentially aiding the decision-making and hypothesis-generating process. In an ideal situation, a human GEM could be constrained with RNAseq data gained from a patient’s tumour, and used to predict therapeutic responses and disease progression, directing treatment decisions and guiding more accurate prognoses. Genome-scale modelling has a greater scope than the direct measurement of metabolic fluxes, as in vitro experiments are limited to measuring only a few dozen reactions in central metabolism^11^. Thereby the hypothesis-generating capability of wet lab methods may be complemented by modelling approaches, providing a worthwhile link between abundant omics data and reaction rate predictions.

Over the past couple of decades, studies into human metabolism have been greatly advanced by GEMs: namely, the Recon series (Recon1, 2 and 3D)^12–14^ and the Human Metabolic Reaction series (HMR1 and 2)^15,16^. In addition to these fundamental models, there have been whole-body GEMs, for example, two complete reconstructions, containing organ- and sex-specific information^17^, from which individual organs could be extracted. Utilising HMR2 as its framework, one project constrained six patient-specific GEMs, which enabled the discovery of anticancer drugs^18^. One of the most recent human GEMs to be developed was the Human1 model, which is an updated and curated version of the Recon and HMR series, demonstrating optimisation such as the removal of duplicated reactions and metabolites, correction of model inconsistencies, and the standardisation of identifiers^19^. However, genome-scale modelling of OC remains limited. One well-cited study reporting the constraining of Recon1 to decipher resistance mechanisms in OC did not provide in vitro validation of growth rates or metabolic predictions, nor was an original GEM or omics integration algorithm developed^20^. A more recent study employed genome-scale modelling to explore morphology-specific metabolic changes within OC, and validated results against existing drug sensitivity data^21^.

There are many existing integration algorithms to constrain these GEMs with omics data, and these have been well-reviewed in the context of flux balance analysis (FBA)^22^. These algorithms differ in the way in which they set reaction bounds, process and segregate expression data prior to its integration, and the variety of ‘omics data which they accept. Some of these algorithms utilise discrete methods, switching reactions on or off according to expression thresholds, for example, iMAT^23^ and GIMME^24^. In contrast, other algorithms use continuous bounds, where expression values are applied directly, such as E-Flux^25^. Some algorithms are able to accept multiple ‘omics types and combine these expression values into reaction constraints, for example, MADE and iMAT accept both gene and protein expression values^23^, whilst other algorithms can incorporate transcriptional regulatory networks, such as PROM^26^. Furthermore, some algorithms extend as far as specifying kinetic parameters—an approach which has been used to understand the role of redox metabolism in radiation-resistant tumours^27^. A systematic and comprehensive evaluation of seven existing ‘omics integration algorithms assessed the performance of enzyme-constrained metabolic models, using comparisons to experimental metabolic flux data^28^. This evaluation was carried out following an influx of integration algorithms in 2012; the work spanned prokaryotic and eukaryotic systems and concluded that no single integration algorithm outperforms all others with respect to its predictive accuracy, and it is recommended that the user selects their algorithm based on their specific applications^28^. Furthermore, it is likely that individual algorithms demonstrate improved potential for target identification, quantitative prediction of baseline metabolic fluxes, or the replication of experimental growth rates compared to other algorithms. The aim of ‘omics integration is to tailor a genome-scale model to be sample-specific, and until one algorithm clearly emerges as being more accurate in its ability to do this, there will be demand for the development of novel methods, directed by experimental data.

All factors considered, this publication presents a novel ‘omics integration algorithm, which accurately tailors the Human1 genome-scale model towards in vitro growth measurements, using experimental growth rates as internal thresholds. The proposed algorithm involves the use of continuous, relative reaction bounds—incorporating normalised transcriptomics data directly. Furthermore, the LGSOC and HGSOC models presented here provide a framework for genetic engineering experiments, which could generate interesting hypotheses for future work, as well as thought-provoking predictions on the difference between LGSOC and HGSOC metabolism.

## Methods

### Cancer cell line encyclopaedia (CCLE) transcriptomics data

Transcriptomics data were obtained from the Broad DepMap Portal, under the DepMap Public 22Q2, titled ‘CCLE_expression_full.csv’ (https://depmap.org/portal/download/all/). Out of tens of primary diseases, this dataset includes 65 ovarian cell lines and 53,949 genes (accessed September 2022). This dataset contains RNAseq TPM gene expression data for all genes using RNA-Seq by expectation-maximization, which has been Log_2_-transformed, and uses a pseudo-count of 1 to avoid negative values. Further normalisation and processing details were described alongside dataset release^29^. As acknowledged by a similar study utilising CCLE data for genome-scale modelling^30^, the relationship between RNA and proteins is complex and not completely understood in the context of its CBM implications, however, there is a moderate positive correlation between gene and protein expression (mean *r* = 0.48)^31^. In addition, although statistical significance in the form of *p*-values was not included in the original publication, a wide range of Pearson correlation scores was reported at −0.56 to 0.989^31^. Furthermore, it was reported that there was the highest correlation between gene and protein expression for molecules involved in the epithelial-mesenchymal transition and cell surface protein-related pathways, whilst the lowest was observed for gene sets with notable protein complexes^31^. Taking all of this into account, and the fact that the correlation of these two ‘omics types for this dataset was reportedly in line with previous studies^31^, indicating reliability, gene expression was used as a proxy for protein expression in this study.

### Cell line annotations and growth conditions

Before models could be built, it was necessary to annotate the CCLE cell lines with their subtypes, so sample groups could be composed. Due to the fact there is contention in literature as to the ‘true’ subtype of some ovarian cell lines, we have chosen to use recent NMF clustering to direct our subtyping^32^, because this project combined both an extensive literature review and a comprehensive NMF clustering workflow to determine the true subtypes of cell lines, demonstrating high diagnostic accuracy on patient-derived models and validating previous classification analyses^32^. This classification informed by Barnes et al. ^32^ reported five distinct histological subtypes for OC based on a CCLE transcriptomics dataset (*n* = 44 cell lines and 6796 genes); this cluster frequency was supported by multiple quality measures. Five distinct subtypes for OC are widely accepted, and these are based on mutational and morphological evidence, clinical presentation and sensitivity to chemotherapy^33^. Furthermore, the subtype classifications assigned by Barnes et al. ^32^ supported a fundamental study into HGSOC by Domcke et al. ^34^, as well as proposing subtype labels for cell lines which previously had no classification, were atypical non-serous or undistinguishable, which gives us a larger pool of classified cell lines to choose from for this analysis.

Media conditions have been reported by Barrentina et al. ^35^, and where these weren’t available for specific cell lines, the source of optimal media, as well as the site of origin, has been referenced in Supplementary Data 3.

### Human1 genome-scale model and Metabolic Atlas

CBM techniques were applied to the Human1 model, which was obtained from the Human-GEM GitHub repository (https://github.com/SysBioChalmers/Human-GEM)^19,36^. The model version used here is titled ‘Human-GEM-annotated.xml’ and was accessed in September 2020. This version includes 13,096 reactions, and of these, 61.4% are annotated with gene-protein-reaction rules, informing the enzyme(s) required for catalysis. These gene-protein-reaction rules are where transcriptomics data has been integrated. Of this annotated portion, there are 3972 ‘or’ rules, which describe isoenzymes; 653 ‘and’ rules, which specify enzyme subunit IDs; 129 ‘andor’ rules which include both isoenzymes and subunits, and the remaining 3282 annotated reactions are ‘one-gene’ rules, which list one single enzyme needed for catalysis. In addition, there are 3628 unique genes in the Human1 model, and of this total, there are only four which are not included in the CCLE transcriptomics dataset (RNF115, RNF128, SLC27A2 and METTL23). The units for the Human1 model are mmol/gDW/h for all reactions, except for biomass production, which is g/gDW/h, therefore the inverse of growth rate corresponds to doubling time. Metabolic Atlas is the accompanying web portal, which was published alongside the Human1 model^19^, and has been used to understand and contextualise cell line-specific results generated from this project.

### CBM and transcriptomics integration workflow

All methods described below, alongside accompanying requirements and dependencies, have been made available on the Github repository ‘repository_to_accompany_paper_2023’ (https://github.com/katemeeson/repository_to_accompany_paper_2023). This method employs FBA, and has been optimised for biomass production, a widely used cellular objective function for the CBM of cancer cells^11^. In order to integrate transcriptomics data, a novel workflow has been developed which uses original code, implementing the MEWpy and COBRApy toolboxes^10,37^. The preliminary step for data integration was to define media conditions, involving exchange and demand reactions, and then normalised gene expression values were integrated with the bulk of the Human1 model, in a cell line-specific manner. For all simulations, the Gurobi solver and Python 3.0 were used^38^. Modelling results can be found in Supplementary Data 2.

### Validating model predictions

In order to validate model predictions, an over-representation analysis (ORA) was performed in WebGestalt, using the ‘pathway’ and KEGG functional database and the ‘genome’ reference set^39^. The input for this search was experimental gene dependency data, which was generated from CRISPR-Cas9 knockout (KO) screens and describes how essential a gene is for growth. This gene dependency dataset was accessed from the CCLE Broad DepMap Portal, under the DepMap Public 22Q2, and titled ‘CRISPR_gene_dependency.csv’. From this dataset, a subset was extracted containing the same cell lines on which CBM was performed, namely, three high-grade (COV318, CAOV3 and OAW28) and two low-grade cell lines (59M and HEYA8), however, the low-grade OV56 cell line was not available. From here, average gene dependencies were calculated for the two low-grade (59M, HEYA8), and three high-grade cell lines (CAOV3, COV318 and OAW28), and then these mean values were compared. The 250 genes which showed the highest increase in dependency in low-grade cell lines (calculated as high-grade subtracted from low-grade gene dependency score) were used as input for the WebGestalt ORA, to predict the overarching pathways which low-grade cell lines might rely on for growth. This input gene set, including 250 genes, was equal to roughly 1.5% of the total 16,383 gene dataset and was equal to the top 18% of all genes with a CRISPR dependency score of 0.75. A dependency score of 0.75 equates to a reduction in cellular growth upon gene knockdown of 75%. If this input gene set was much smaller than 250 genes, the number of biological functions reported by the ORA would likely reduce, and the sensitivity of biological conclusions could be lost. In addition, if this input dataset was smaller, the statistical significance reported by WebGestalt could be reduced as there might be fewer genes associated with an over-represented pathway. On the other hand, a smaller input dataset could also mean less ‘noise’ and improve the specificity of the ORA, therefore, the size of the input dataset is an important variable to consider. The accompanying notebook for this method can be accessed at https://github.com/katemeeson/repository_to_accompany_paper_2023.

Results were also validated by comparing the growth effect of in silico KO simulations set up using COBRApy^10^, with the CRISPR-Cas9 experimental data described above. A ratio of growth after single gene deletion over optimal growth was calculated and recorded for all 3,628 genes in the model. From here, genes present in both the CRISPR-Cas9 dataset and model simulations (*n* = 3465) had their gene dependency score and KO simulation growth ratio correlated. Due to the expected linearity between in silico growth ratio and in vitro gene dependency and given the dependency score was originally calculated as a function of growth, the Pearson correlation coefficient was used. Following an analysis of all 3465 genes, a subset of genes with predicted high essentiality were correlated, namely those with a growth ratio of less than 1.0. The accompanying notebook for this method can be accessed via https://github.com/katemeeson/repository_to_accompany_paper_2023.

## Results

### Determining the appropriate range of media constraints

**Fig. 1:**
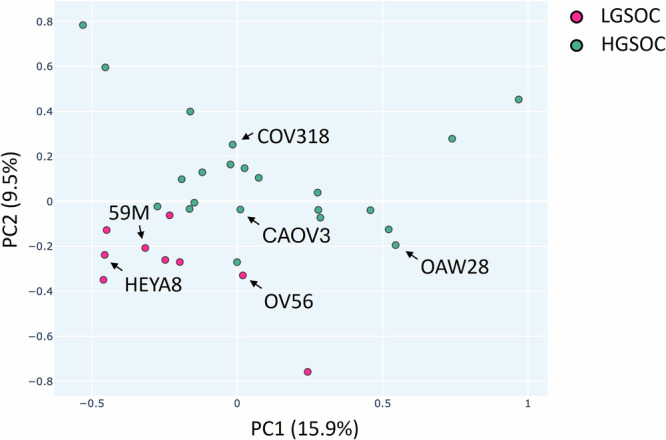
Visualisation of selected cell lines. 2D Principal components analysis visualising the spread of the gene expression (CCLE) of LGSOC and HGSOC cell lines, with chosen cell lines highlighted. ‘True’ labels were assigned according to Barnes et al. ^32^. Teal are high-grade, and pink are LGSOC.

ecGEMs were constructed for three low-grade (59M, HEYA8 and OV56) and three high-grade serous ovarian cell lines (CAOV3, COV318 and OAW28). To direct the selection of cell lines for sample groups, the entire subset of CCLE LGSOC and HGSOC cell lines was visualised using PCA and labelled according to their subtype (Fig. 1), media conditions and site of origin (Supplementary Fig. 1). The purpose of this visualisation was to deduce whether or not the contrasting media conditions and site of origin directly affected gene expression, and if so, the acceptable range of these variables for the cell lines to be compared in this study. The constrained models had their media completely standardised to DMEM, then one single cell line (CAOV3) had its media systematically changed to assess how this affected the spread of points on PCA (Supplementary Fig. 2). These comparisons showed that whilst DMEM remained a main component of the media, the spread of metabolic flux results did not change.

### Defining media constraints within the Human1 model

Prior to the integration of transcriptomics data, the media conditions were defined for each cell line model (Supplementary Table 1). Media conditions were integrated using the MEWpy ‘get_simulator’ function, setting the final media dictionary as the ‘envcond’ argument^37^. A limitation of defining media for human GEMs is the inclusion of foetal bovine serum (FBS), which is a complex, difficult-to-define solution. Therefore, FBS components were estimated by reopening specific reactions according to predicted essentiality (relaxing bounds back to original −1000 mmol/gDW/h or 0 mmol/gDW/h and +1000 mmol/gDW/h); for example, the exchange reaction ‘MAR09107’, which imports haem into the cytosol, was reopened in the media dictionary for the HEYA8 low-grade cell line. Haem is suggested to be present in FBS preparations at a concentration between 1.1 μM and 2.0 μM^40^, which validates the assumption that this metabolite is imported into the cellular models, despite it not being specified in DMEM ingredients. Final copies of media-constrained models were saved and utilised in the transcriptomics integration stages described below (details available on https://github.com/katemeeson/repository_to_accompany_paper_2023).

### Integrating transcriptomics data with Human1 gene-protein-reaction rules

The novel transcriptomics integration method presented here is based on the incorporation of expression data based on cross-reference with Human1 gene-protein-reaction rules, which define the enzymes and their genes required for reaction catalysis. Every gene reaction rule contained within Human1 has a lower and upper bound, with default excess reaction rate limits of −1000 mmol/gDW/h and +1000 mmol/gDW/h, or 0 mmol/gDW/h and 1000 mmol/gDW/h, based on reaction reversibility. Whether or not the expression data is applied directly, as a sum or as a minimum value, depends on the category of the reaction rule (Table 1). Reaction rules using the ‘andor’ term have not been included in this integration method, due to the fact they only represent 1.6% of the total annotated portion of the model.

**Table 1:** Method of rule-dependent expression data integration

Human1 reaction rule category	Gene-protein-reaction rule schematic	Method of integrating expression data
One-gene	$A + B\mathop{\rightarrow}\limits^{E}C$	Lower bound = −*E* or 0 Upper bound = +*E*
‘or’ (isoenzymes)	$A + B\mathop{\longrightarrow}\limits^{{E}_{1}\,or\,{E}_{2}}C$	Lower bound = (−*E*_1_ + −*E*_2_ … + −*E*_x_) or 0 Upper bound = *E*_1_ + E_2_ … + *E*_x_
‘and’ (enzyme subunits)	$A + B\mathop{\longrightarrow}\limits^{{E}_{a}\,and\,{{E}}_{b}}C$	Lower bound = −1 × min (*E*_a_, *E*_b_,… *E*_x_) or 0 Upper bound = min (*E*_a_, *E*_b_,… *E*_x_)

If the Human1 reaction rule specifies a single gene, then the normalised expression data can be used directly as the reaction bounds. If an ‘or’ term is specified (and no ‘and’ term), then the normalised abundances of either isoenzyme can be summed, as the probability of the reaction being catalysed has been assumed to be directly proportional to the cumulative amount of compatible isoenzymes. If there is an ‘and’ term specified (and no ‘or’ term), then the minimum expression value of all available subunits is taken as the reaction bounds, as both subunits must be present for the reaction to be catalysed. *A* and *B* denote reactants; *C* denotes product; *E* denotes reaction-specific enzyme; *E*_1_ and *E*_2_ denote isoenzymes, and *E*_a_ and *E*_b_ denote enzyme subunits. Where ‘−*E* or 0’ (mmol/gDW/h) has been specified for the lower bound in the final column, this refers to a reaction which is reversible (−*E* mmol/gDW/h) or unidirectional (0 mmol/gDW/h lower bound), respectively.

**Fig. 2:**
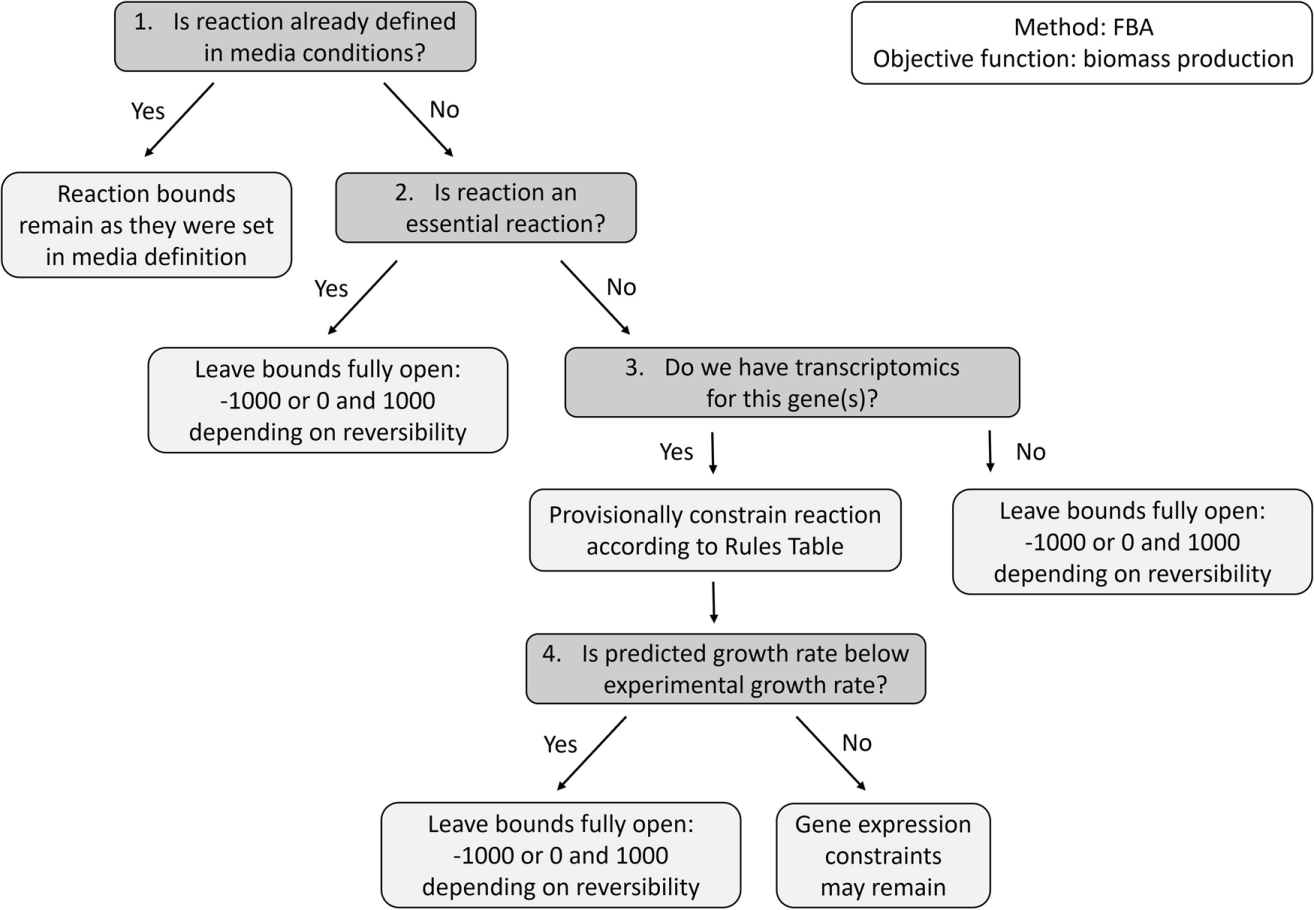
The transcriptomics integration workflow. First, the algorithm checks whether the target reaction has already been defined in the media conditions, and if so, it leaves the reaction bounds as they are. Next, if the target reaction has been defined as essential according to a MEWpy simulation, the algorithm leaves the reaction bounds as they were originally (−1000 mmol/gDW/h or 0 mmol/gDW/h and 1000 mmol/gDW/h, depending on reversibility). Then, if the gene(s) for this reaction are contained within the input transcriptomics dataset, then the reaction is provisionally constrained according to the corresponding reaction rule described in Table 1. Finally, FBA is run and if the solution is not below the experimentally defined minimum growth rate, the constraints may remain, however, if a growth rate is predicted which is below this experimental threshold, then the reaction’s bounds are relaxed.

Transcriptomics data was integrated by reopening and constraining reactions, based on the individual gene’s inclusion in the CCLE expression dataset, media conditions, essentiality predictions and experimentally defined growth thresholds. The workflow for transcriptomics integration has been summarised as a decision tree (Fig. 2). To begin with, the algorithm asked whether the reaction had already been defined in media conditions, and whether it was essential. If the answer to these queries was negative, then the algorithm searched the input transcriptomics data for the presence of a gene(s) described in the gene-protein-reaction rule. If the gene(s) for this reaction were present in the input dataset, then the reaction was provisionally constrained according to gene expression (Table 1). It has been reported that gene and protein expression within the CCLE dataset have low correlation^31^, and also that many omics integration algorithms, such as iMAT, do not accurately predict growth rates as they do not account for metabolic deviation or adaptation from a transcriptomics signature^41^. Therefore, here, experimental growth rates have been used as a limit for model-predicted growth, and we reopened any reactions which forced growth rates beyond this limit. This means experimental growth thresholds are serving to estimate where gene expression does not accurately represent enzyme abundance and predict experimentally derived growth rates. This growth-directed ‘omics integration aims to compensate for poor correlation between the expression of some genes and proteins, by comparing growth predictions to experimental measurements with each subsequent expression value which is integrated, therefore this should be an indicator of gene and protein expression correlation. However, in general, we advise future algorithm users that a Pearson correlation value of 0.5 (and a significant associated *p*-value) should indicate a good correlation.

### Using experimental maxima to tailor model-predicted growth rates

**Fig. 3:**
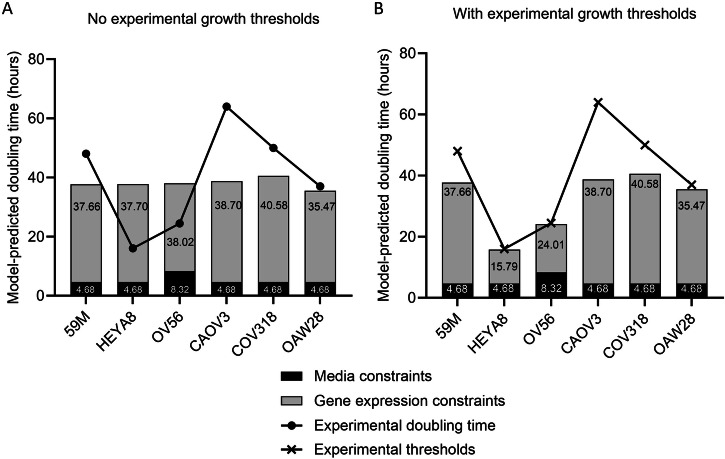
Visualisation of experimental growth thresholds. Experimental doubling times and their origin have been shared in Supplementary Table 2. **A** Media constraints were applied to unconstrained Human1 using MEWpy (black bars), and then gene expression was integrated (grey bars) using our novel integration algorithm. Here, no growth thresholds were applied. **B** Experimental growth measurements have been translated to thresholds for model growth predictions.

Prior to data integration, the flux through biomass production was approximately 187 g/gDW/h. Following the application of media constraints described above, the doubling time dropped to between 4.5 h and 9 h for every model (the equivalent of between 0.11 g/gDW/h and 0.22 g/gDW/h) (Fig. 3). Although a lot more realistic than the original, unconstrained solution, these doubling times were still considered too dissimilar to experimental values and needed supplementing with gene expression constraints to reach a more realistic value and construct cell-line-specific models.

Once gene expression values had further constrained models, FBA-predicted growths were compared, both before and after the application of experimental growths as model thresholds. Before the application of thresholds, models predicted a maximum doubling time of between approximately 35 h and 41 h, across all cell lines (Fig. 3A). Experimental thresholds were most effective at tailoring model growth predictions towards experimental values in the quicker proliferating cell lines, HEYA8 and OV56 (Fig. 3B). On the other hand, no reactions had to be reopened for 59M, CAOV3, COV318 and OAW28, because the FBA-predicted doubling time never reached the experimental limit. The fact that no reactions were reopened in these four models means that constrained reaction bounds directly reflect gene expression data and media conditions. It has been concluded that using the experimental growth values as model thresholds helped generate experimentally representative metabolic predictions regarding biomass production. Thus, this investigation was allowed to proceed to flux analysis.

An analysis visualising the FBA-predicted growth before any model constraints, after media constraints, and finally after gene expression and experimental growth threshold constraints have been included in Supplementary Data 1 to illustrate the impact of ‘omics integration (Supplementary Fig. 3).

### Reopening reactions to satisfy experimental growth thresholds does not skew downstream metabolic predictions

Of the 13,096 reactions contained within the Human1 model, only six unique reactions had to have their constraints relaxed (bounds forced to −1000 mmol/gDW/h or 0 mmol/gDW/h, and +1000 mmol/gDW/h, depending on reversibility) across the cell lines to satisfy experimental growth thresholds (Table 2). Therefore, the majority of fluxes analysed were representative of gene expression data. Several reactions were reopened in HEYA8 and OV56, to bring the model-predicted doubling time back within the experimental thresholds (Table 2). For HEYA8, there were four reactions reopened: three of these were involved in oxidative phosphorylation, whilst reaction ID MAR07663 occurred within the biotin metabolism subsystem. Concerning OV56, there were three reactions reopened: two of which represented a stage of oxidative phosphorylation, and the remaining MAR00786 which was involved in sphingolipid metabolism. There are only ten reactions in the Human1 model which constitute oxidative phosphorylation, meaning that a large proportion of reaction bounds within this subsystem have been reopened to excess (−1000 mmol/gDW/h or 0 mmol/gDW/h, and 1000 mmol/gDW/h), and in turn are not directly representative of cell line-specific gene expression. Flux predictions for these reopened reactions have been listed in Supplementary Data 2, and show that as expected, there were increased flux predictions for the cell lines for which these reactions were reopened. Therefore, although these relaxed reaction rate boundaries do not necessarily mean the FBA-predicted reaction flux is not realistic, conclusions made during this investigation will not involve oxidative phosphorylation.

**Table 2:** Reactions reopened to keep model-predicted doubling times below experimental thresholds

Cell line	Reactions reopened	Reaction description
59M	N/A	
HEYA8	MAR06916	ADP[m] + 3 H^+^[i] + Pi[m] → ATP[m] + 2 H^+^[m] + H_2_O[m]
	MAR06918	2 H^+^[m] + 2 ferricytochrome C[m] + ubiquinol[m] → 4 H^+^[i] + 2 ferrocytochrome C[m] + ubiquinone[m]
	MAR13081	7.92 H^+^[m] + O_2_[m] + 4 ferrocytochrome C[m] → 4 H^+^[i] + 1.96H_2_O[m] + 0.02O_2_^−^[m] + 4 ferricytochrome C[m]
	MAR07663	H_2_O[e] + biocytin[e] → biotin[e] + lysine[e]
OV56	MAR06916	ADP[m] + 3 H^+^[i] + Pi[m] → ATP[m] + 2 H^+^[m] + H_2_O[m]
	MAR06921	5 H^+^[m] + NADH[m] + ubiquinone[m] → 4 H^+^[i] + NAD^+^[m] + ubiquinol[m]
	MAR00786	H_2_O[l] + LacCer pool[l] → galactose[l] + glucosylceramide pool[l]
CAOV3	N/A	
COV318	N/A	
OAW28	N/A	

For 59M, CAOV3, COV318 and OAW28 there were no reactions reopened. For HEYA8 and OV56 there were 4 and 3 reactions reopened, respectively. Reaction IDs correspond to those listed in Metabolic Atlas^19^, where additional reaction annotations can be found.

The fluxes through reactions immediately downstream of the reopened reactions (Table 2) have been described in Supplementary Data 2, to check if the reopening of model reactions to satisfy experimentally predicted growth rates skewed overall flux results. Not all downstream fluxes were analysed, as some reopened reactions produced metabolites involved in hundreds of reactions, for example, MAR06916, which produces mitochondrial ATP, is connected to 1114 reactions via its products. Results show that in general, there is no overarching trend of downstream reactions to match the flux pattern of those reopened immediately upstream. For example, MAR06918 was reopened in HEYA8, and is connected to twenty-eight reactions immediately downstream via the metabolites ferricytochrome C and ubiquinone. Of these downstream reactions, only eight showed an increased flux in HEYA8, whilst the remaining twenty reactions show either zero flux across all cell lines or are increased in cell lines other than HEYA8. Furthermore, another reaction reopened in HEYA8 was MAR13081, and of the five reactions immediately downstream of MAR13081, four either showed zero flux across all cell lines or had a higher predicted flux in another cell line than HEYA8. This indicates that the relaxing of a few reactions’ bounds to tailor growth rates towards experimental measurements does not skew the overall metabolic flux through cell line-specific models, and fluxes could be more dependent on the transcriptomics measurement—or lack of—which constrains them specifically, or the flux through other upstream reactions.

### Identifying subtype-specific differences in central metabolism

Following analysis of growth predictions, individual reaction fluxes were compared. In order to compare fluxes between low- and high-grade samples, we specified an initial criterion for difference between models, comprising multiple factors: there must be a 10% increase or decrease between the mean flux value for the given reaction; at least one flux value above 0.5 mmol/gDW/h for unidirectional reactions, or −0.5 mmol/gDW/h for reversible reactions, or reaction flux has changed direction between low- and high-grade models. Of the 13,096 total reactions, 25.5% of the non-exchange/demand reactions had a predicted non-zero flux through at least one of the cell lines. This meant 74.5% of metabolic activity was predicted to be switched off across all of the cell line-specific models. Of the non-zero subset, there were 1081 reactions which satisfied the criteria for difference—corresponding to 8.25% of active metabolism which initial criteria suggested may display a subtype-specific flux. This initial subset of reactions has been provided in Supplementary Data 2.

The next step of this analysis was to gain an overview of the central metabolic subsystems which were enriched by these 1081 potentially differentially-regulated reactions. Central metabolism was broadly defined according to the ‘global and overview maps’ listed in KEGG (*KEGG PATHWAY* Database, 2022)^42–44^. Specifically, the following subsystems were included in this subset: nucleotide metabolism, lipid metabolism, fatty acid metabolism, carnitine shuttle, central carbon metabolism (including glycolysis/gluconeogenesis, tricarboxylic acid and glyoxylate/dicarboxylate metabolism and the pentose phosphate pathway), and amino acid metabolism. A database annotation search was performed in Metabolic Atlas, and of the initial 1081-reaction subset described above, 710 were annotated with gene-protein-reaction rules, specifying the enzyme(s) which were required for catalysis; of these, there were 313 reactions belonging to central metabolic processes. Nucleotide metabolism was the subsystem containing the greatest number of reactions satisfying our initial criteria for subtype-specific flux, with 115 reactions showing differences in their mean fluxes (Table 3).

**Table 3:** Overview of the central metabolic subsystems containing top model-predicted differences

Metabolic subsystem	Number of reactions with a 10% change in mean flux between low- and high-grade models	Number of unique enzymes controlling these reactions
Nucleotide metabolism	115	61
Amino acid metabolism	52	68
Carnitine shuttle	47	31
Fatty acid metabolism	39	24
Lipid metabolism	27	94
Glycolysis/gluconeogenesis	11	29
Tricarboxylic acid cycle and glyoxylate/dicarboxylate metabolism	11	14
Pentose phosphate pathway	11	18

Reaction count and unique enzymes involved in the 313 central metabolic reactions, were initially predicted to have different fluxes (up- or downregulated) between low- and high-grade models. A full list of reaction IDs has been presented in Supplementary Data 2.

**Fig. 4:**
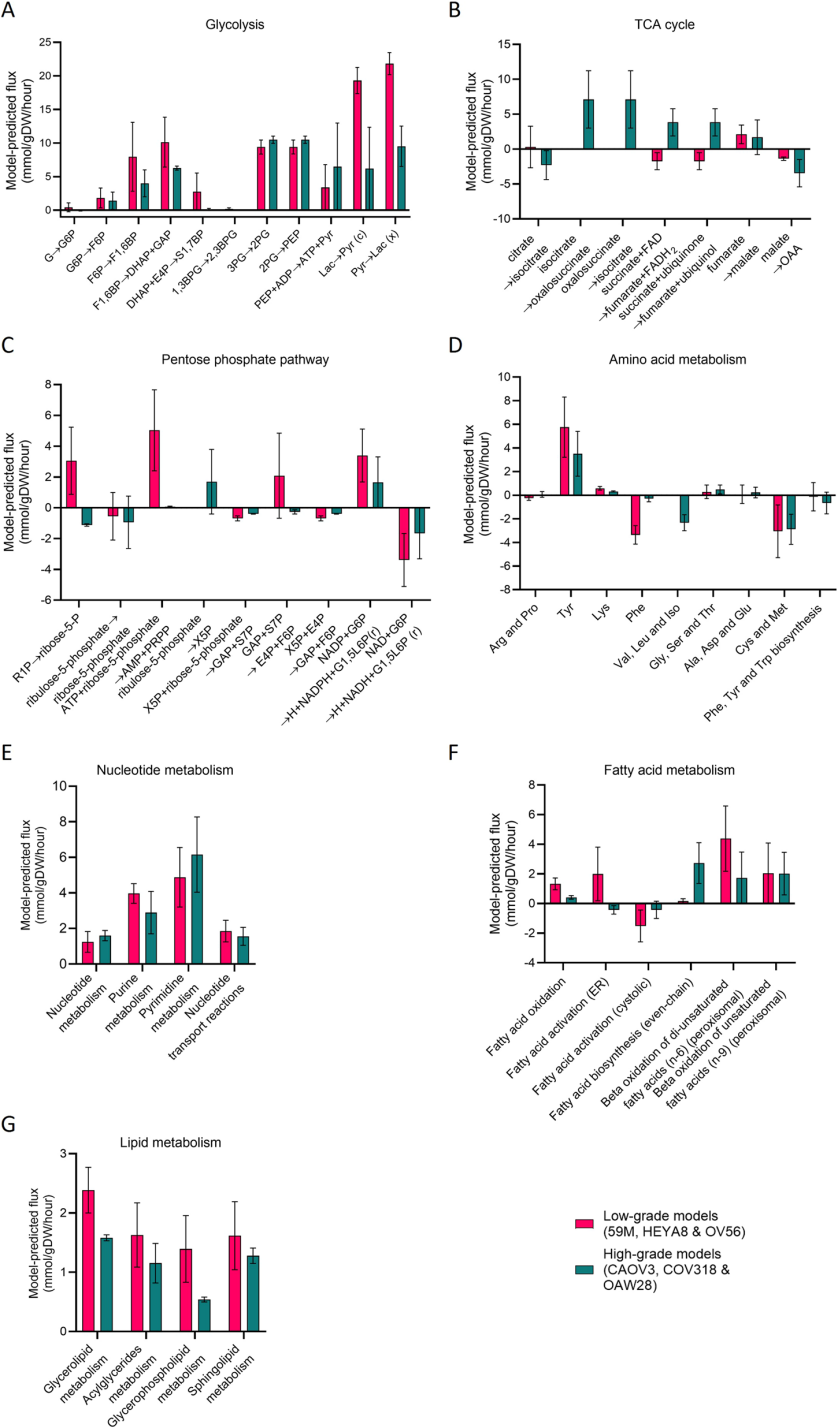
Reaction-level flux differences between low- and high-grade ovarian models. **A** Glycolysis subsystem. Reactions in glycolysis which models have predicted to be differentially regulated across between low- and high-grade serous ovarian cancer. **B** TCA cycle subsystem, low- versus high-grade metabolic predictions. **C** Pentose phosphate pathway subsystem, low- versus high-grade metabolic predictions. **D** Amino acid metabolism subsystem, low- versus high-grade metabolic predictions. **E** Nucleotide metabolism subsystem, low- versus high-grade metabolic predictions. **F** Fatty acid metabolism subsystem, low- versus high-grade metabolic predictions. **G** Lipid metabolism subsystem, low- versus high-grade metabolic predictions. Plots to show model-predicted fluxes (mmol/gDW/h) through central metabolism, averaged across *n* = 3 models per subtype. Some of these reactions are reversible, however, for clarity of visualisation, the direction shown is such that positive reaction flux corresponds to the simplified reaction equation on the x-axis. The colour of the bar corresponds to the subtype, as indicated in the key. The mean value is plotted, and the standard error margin is shown on each bar. Some subsystems, e.g. amino acid metabolism, have had multiple reaction fluxes grouped into subcategories and averaged; individual reaction fluxes are available in Supplementary Data 3. Compartments: all glycolytic reactions are cytosol unless specified (‘c’ = cytosol; ‘x’ = peroxisome); all TCA cycle occurs in mitochondria; all pentose phosphate pathways in cytosol unless specified (‘r’ = ribosome); amino acid metabolism, nucleotide metabolism, fatty acid metabolism and lipid metabolism occurs across a range of subsystems. (G glucose, G6P glucose-6-phosphate, F6P fructose-6-phosphate, F1,6BP fructose-1,6-bisphosphate, DHAP dihydroxyacetone phosphate, GAP glyceraldehyde 3-phosphate, E4P erythrose-4-phosphate, S1,7BP sepoheptulose-1,7-bisphosphate, 1,3BPG 1,3-bisphosphoglycerate, 2,3BPG 2,3-bisphosphoglycerate, 3PG 3-phosphoglycerate, 2PG 2-phosphoglycerate, PEP phosphoenolpyruvate, Pyr pyruvate, Lac l-lactate, OAA, oxaloacetate, R1P ribose-1-phosphate, PRPP phosphoribosyl pyrophosphate, X5P xylulose 5-phosphate, S7P sedoheptulose 7-phosphate, G1,5L6P glucono-1,5-lactone-6-phosphate).

The metabolic subsystems described above have been analysed at the individual reaction level, to further understand what subtype-specific differences models might predict (Fig. 4). Results have been organised to present model-predicted flux through individual reactions across central subsystems and smaller branches of these subsystems according to Metabolic Atlas^19^ (Fig. 4). For individual and grouped reactions, flux has been averaged across (*n* = 3) low- and (*n* = 3) high-grade models to help reveal metabolic patterns which are consistent across different models of the same subtype.

Initially, there were eleven glycolytic reactions included in the subset of 313 reactions, which satisfied our initial criteria for differential regulation. Models indicate that the oxidation of lactate to pyruvate in the cytosol (MAR04388) is likely to be increased in low- compared to high-grade models, as indicated by a mean flux value which is over 3× higher, and non-overlapping standard error (Fig. 4A). Similarly, low-grade models predict an increased rate of the reduction of pyruvate to lactate via the peroxisomal lactate-pyruvate redox shuttle (MAR04281), which regenerates NAD^+^ for β-oxidation^45^ (Fig. 4A).

There are eleven reactions across the TCA cycle which have a predicted reaction flux at least 10% different between low- and high-grade models (Fig. 4B). A futile cycle is a set of two simultaneous reactions, which flow in opposite directions, and result in no net gain or loss of reactants or products^46^. Although potentially an artefact of FBA, futile cycles are a true phenomenon in biology. Models suggest the presence of a futile cycle between reactions MAR04588 and MAR04586, which involve the dehydrogenation of isocitrate to oxalosuccinate and the re-conversion of this back to isocitrate, respectively (Fig. 4B). On a separate note, models predict some upregulation of the TCA cycle in high-grade models, specifically MAR08743 and MAR04652, which catalyse the reversible oxidation of succinate to fumarate via reduction of FAD and ubiquinone, respectively (Fig. 4B). These TCA reactions do not occur in a futile cycle, because the production of fumarate via MAR04652 occurs at a slightly higher rate than MAR08743, which reduces fumarate to succinate.

Initially, there were eleven reactions in the pentose phosphate pathway which were predicted to have at least 10% change in flux between low- and high-grade models (Fig. 4C). Results indicate that of these, there were four which showed the strongest subtype-specific regulation (Fig. 4C). Ribose-1-phosphate comes from nucleotide metabolism and is isomerised by phosphoglucomutase to generate ribose-5-phosphate (MAR04354), which feeds into the non-oxidative phase of the pentose phosphate pathway. Flux analysis predicts that this process is upregulated in the forward direction in the low-grade models (Fig. 4B). Furthermore, models predict the upregulation of reactions MAR04501 and MAR04404 in low-grade models, which would lead to the increased production of xylulose-5-phosphate and ribose-5-phosphate, and xylulose-5-phosphate and erythrose-4-phosphate, respectively (Fig. 4C). Finally, models predict that the conversion of ribose-5-phosphate to phosphoribosyl diphosphate (MAR04052) is predicted to be upregulated in low-grade models (Fig. 4B).

Regarding amino acid metabolism, there is an increase in the predicted fluxes across both lysine and phenylalanine metabolism in low-grade models (Fig. 4D). Considering high-grade models, there is an increased average predicted flux across valine, leucine and isoleucine reactions (Fig. 4D). Amino acid metabolism connects to nucleotide metabolism via metabolites such as fumarate, which feed into nucleotide, purine and pyrimidine synthesis. However, despite results indicating amino acid metabolism as being differentially regulated between low- and high-grade models, results suggest no subtype-specific patterns of nucleotide metabolism (Fig. 4E).

Fatty acid metabolism can be subdivided into its reaction categories, namely oxidation, activation and biosynthesis (Fig. 4F). Interestingly, results indicate potential low- and high-grade signatures within this subsystem. Across low-grade models, reactions involved in fatty acid oxidation and activation (in the endoplasmic reticular) are upregulated (Fig. 4F). In contrast, there is an increased mean flux in high-grade models through fatty acid biosynthesis, to produce even-chain fatty acids (Fig. 4F).

Finally, lipid metabolism may be divided into multiple subcategories, depending on the class of lipid molecule being metabolised. Flux analysis predicts a higher flux across reactions of glycerolipid and glycerophospholipid metabolism for low-grade models, each of which contains two and six reactions upregulated in low-grade models, respectively (Fig. 4G).

### A proposed mechanism for low- vs HGSOC metabolism

**Fig. 5:**
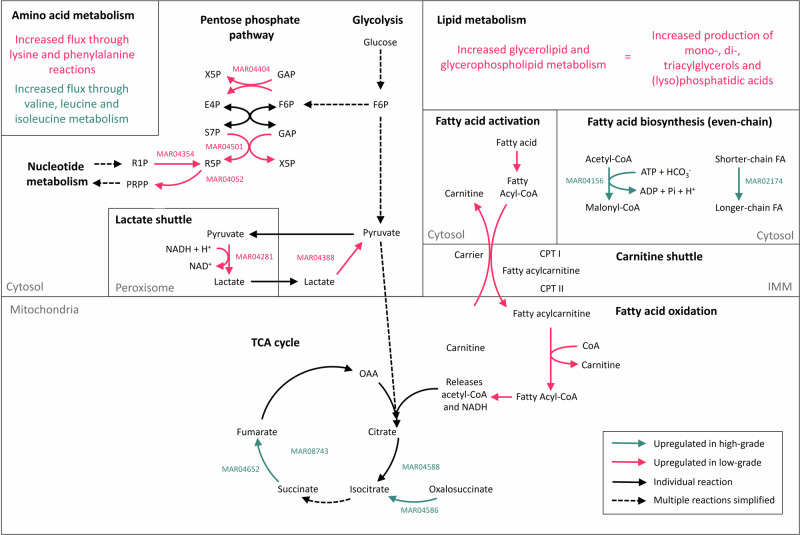
Proposed mechanism for model-predicted low- and HGSOC metabolic differences. Compartments have been labelled (and where they are not labelled, it is because there are multiple compartments per subsystem), and the colour of the arrow denotes upregulation in LGSOC (pink) and HGSOC (teal) models. Human1 reaction IDs annotated where an individual reaction has been described. A solid arrow corresponds to a single reaction or general one-step conversion, and a dotted arrow denotes multiple reactions simplified. (X5P xylulose-5-phosphate, GAP glyceraldehyde 3-phosphate, E4P erythrose-4-phosphate, F6P fructose-6-phosphate, S7P sedoheptulose 7-phosphate, R5P ribose-5-phosphate, R1P ribose-1-phosphate, PRPP phosphoribosyl pyrophosphate, FA fatty acid, CPT I/II carnitine palmitoyltransferase I/II, IMM inner mitochondrial membrane, OAA oxaloacetate).

CBM has predicted multiple subtype-specific differences which might exist for serous OC, and these have been summarised as a proposed mechanism (Fig. 5). Overall, results indicate an upregulation of the biosynthesis of even-chain fatty acids in high-grade models, as well as increased flux through valine, leucine and isoleucine metabolism. In addition, results highlighted a futile cycle within the TCA cycle of high-grade models - comprising the interconversion of isocitrate and oxalosuccinate in the TCA cycle.

However, what was clearer, were the multiple reactions and subsystems suggested to be upregulated in low-grade models. Results indicate an increased reaction flux beginning with fatty acid activation in the cytosol, passing via the carnitine shuttle into the mitochondria, where fatty acid oxidation occurs. As a result, there could be an increased rate of release of acetyl-CoA and NADH which feed into the TCA cycle (Fig. 5). In low-grade models, there is also a predicted increased rate of metabolic flow through the non-oxidative phase of the pentose phosphate pathway, leading to an increase in production of ribose-5-phosphate, which is then converted to phosphoribosyl pyrophosphate—an intermediate in the production of pyrimidine and purine nucleotides. Results predict that there is an increased rate of oxidation of lactate to pyruvate in the cytosol of low-grade cell lines, as well as an increased reduction of pyruvate to lactate via the peroxisomal lactate-pyruvate redox shuttle, and this regenerates NAD^+^ which may feed into fatty acid oxidation (Fig. 5). Flux analysis also predicts an increase in flux through glycerolipid and glycerophospholipid metabolism, which contributes to the production of mono-, di- and triacylglycerols, as well as phosphatidic and lysophosphatidic acids (Fig. 5).

### Gene dependency experimental data supports model predictions

In order to validate model predictions, an ORA was performed in WebGestalt, using experimental CCLE CRISPR gene dependency data as an input^29^. This analysis suggested the metabolic subsystems which low-grade cell lines rely on for growth. Of the mean gene dependency scores (low-grade average, and high-grade average), the 250 gene IDs which showed the highest dependency in low-grade cell lines were used as input for the ORA. Of these 250 IDs, 246 could be mapped to Entrez gene IDs, and 116 of these IDs could be annotated with functional categories in WebGestalt. The most enriched pathway was the pentose phosphate pathway, with an FDR value of 0.0099033, a *p*-value of 8.7 × 10^−5^ and an enrichment ratio of 10.73. There were five genes identified from the mapped input which were involved in the pentose phosphate pathway, including: glucose-6-phosphate dehydrogenase (G6PD), glucose-6-phosphate isomerase, 6-phosphogluconolactonase, ribose 5-phosphate isomerase A and transketolase (TKT).

Assuming that genes with high dependency scores upregulate their corresponding model reactions to a greater extent, the CCLE gene dependency data agrees with model predictions. To begin with, G6PD catalyses the oxidation of glucose-6-phosphate to glucono-1,5-lactone-6-phosphate (MAR04304), a reaction with a higher average flux in low-grade models (Supplementary Data 2). Similarly, the two TKT-catalysed reactions of the Human1 model (MAR04501 and MAR04404) showed upregulation in low-grade models, which matched the relatively higher gene dependency scores listed on the CCLE.

As well as the ORA, we performed an in silico KO simulation to determine whether models could replicate the growth patterns reported in the CCLE CRISPR growth dependency dataset. Here, single genes were knocked out, and growth was calculated before and after, to show which genes were essential for cell lines to reach optimal growth rates. This analysis was performed on the essential genes of the ovarian models, namely those which had a predicted effect on biomass production once knocked out. This left between 263 and 391 essential genes predicted per cell line, and there was a general pattern of negative correlation (Pearson correlation coefficients between −0.343 and −0.462; https://github.com/katemeeson/repository_to_accompany_paper_2023), showing that as experimental growth dependency scores increased, model simulations predicted that KO of the same gene would be increasingly detrimental for cell growth.

Considered together, these validations show the models can predict features of metabolism which support the growth of OC, in a subtype-specific manner, and in silico simulations show agreement with experimental gene dependency data, for predicted essential genes.

## Discussion

Results presented here indicate a difference in the metabolism of low- and HGSOC, with analysis predicting that of the active metabolism (roughly one-quarter of total reactions), there are differences across multiple areas of central metabolism. These individual reactions and subsystem-level differences can be connected to various clinical phenotypes observed for low- and high-grade OC, such as chemoresistance and metastatic and proliferative potential.

Cell line-specific models predict that the conversion of lactate to pyruvate in the cytosol (MAR04388), and subsequent reduction of this molecule back to lactate in the peroxisome (MAR04281) is upregulated in low-grade models. When these observations are put in the context of wider metabolic pathways, they provide evidence for increased activity across the peroxisomal lactate shuttle. During aerobic glycolysis, glucose is converted to pyruvate in the cytosol, which can then be passed into the peroxisomes via MCT2 transporters^45^. Here, pyruvate is reduced to lactate via peroxisomal lactate dehydrogenase, which is then transported into the cytosol via MCT2, and oxidised back into pyruvate to complete the cycle^45^. During the reduction of pyruvate to lactate in the peroxisomes, NAD^+^ is generated, which feeds into fatty acid oxidation: another pathway which is upregulated in low-grade models (Figs. 4 and 5). As well as supporting fatty acid oxidation, the peroxisomal lactate shuttle maintains the redox balance of the cell^45^.

Genome-scale modelling identified potential subtype-specific signatures within OC, and these can be linked to existing biomarkers and treatment strategies. For example, resistance to platinum-based chemotherapies is a major obstacle in the treatment of low-grade ovarian tumours, and research suggests that lonidamine—a drug which strongly inhibits lactate transport—increases cisplatin toxicity in both platinum-sensitive and resistant tumours^47^. This supports the observation that the lactate shuttle is upregulated in low-grade ovarian models. In addition, fatty acid oxidation was upregulated in low-grade models, and research connects the inhibition of fatty acid oxidation to improved sensitivity of chemoresistant lung adenocarcinoma and chronic lymphocytic leukaemia to paclitaxel^48^.

Modelling also predicted the upregulation of the pentose phosphate pathway in low-grade tumours, in particular, the non-oxidative phase and production of PRPP had an increased flux. Current research suggests that in chemoresistant breast tumours, there is activation of the P13/Akt, Ras and Src signalling pathways, which supports the upregulation of the PPP^49^. Results presented here indicate that a similar metabolic signature could exist for low-grade tumours and explain their resistance to chemotherapy. Furthermore, the non-oxidative phase of the pentose phosphate pathway is essential for maintaining proper redox balance in cells, and increased defence against reactive oxidative species has been described as responsible for treatment resistance^50,51^. Therefore, redox homoeostasis is a popular topic for therapeutic research, and has been explored in OC. For example, various molecules have been found to be overexpressed in OC and linked to poor therapeutic response, such as glutathione, peroxiredoxin and glutathione peroxidases^50^. The upregulation of the PPP in ovarian tumours as a mechanism of cancer progression agrees with current research, which shows that inhibition of this pathway, via 6-aminonicotinamide and dichloroacetate, was able to limit cellular proliferation^52^.

Another feature of low-grade models was the upregulation of lipid metabolism, specifically glycerolipid and glycerophospholipid pathways (Figs. 4 and 5). Increased levels of triacylglycerols have been found in the ascites of ID8-bearing murine tumours, as a consequence of the overexpression of FASN, and have been linked to an immunosuppressive phenotype^48^. In addition, ovarian tumours are reported to rely more heavily on de novo synthesis of fatty acids rather than exogenous, and a key part of this pathway is the esterification of lipid droplets for storage^49^.

A clinical phenotype which distinguishes tumour subtypes is the increased proliferative and invasive potential of high-grade OC, and this can be explained with modelling results. High-grade models predicted an upregulation of fatty acid biosynthesis (MAR04156 and MAR02174; Fig. 5). Cells synthesise long-chain fatty acids as building blocks for the cell membrane, as well as being metabolic intermediates, an important source of energy and immune signalling molecules^53^. FASN regulates one of the reactions shown to be upregulated in high-grade models (MAR02174), and the expression of this enzyme has already been correlated with the grade of clear-cell OC^48^, which implies a similar association could exist for high-grade tumours. Furthermore, there have been multiple inhibitors developed against FASN, for example, cerulenin, which increases survival rates and improves anti-tumour immune response in xenograft ovarian models, and orlistat—a drug which demonstrates synergy with cisplatin in vivo. In addition, modelling has predicted ACC to exert a subtype-specific effect in ovarian models, via the regulation of MAR04156. ACC catalyses the carboxylation of acetyl-CoA to malonyl-CoA, and this enzyme has been studied in the context of OC. Acetyl-CoA itself is elevated in OC, and is acted upon by ACC, therefore research has focused on the inhibition of ACC as a strategy to treat tumour progression^54^. For example, there is an allosteric inhibitor (TOFA) which has brought upon G0/G1 cell cycle arrest and apoptosis in OC cells^54^. The position of FASN and ACC as existing drug targets exemplifies the significance of high-grade modelling results presented here.

Despite the translational potential of cell line-specific models, there remain some limitations of genome-scale modelling that may be overcome with future in silico developments. For instance, FBA requires an objective function to be defined, meaning all flow-through reactions in the model are optimised for one cellular task. Biomass production, in general, is a good objective function for modelling cancer cells, due to replicative immortality being a hallmark of cancer. The definition of the objective function affects gene essentiality analysis, which categorises genes as essential if, upon their deletion, biomass production is reduced to zero. However, as we know, human cells are specialised for a range of metabolic tasks, as shown by a discussion of the definition of gene essentiality accompanying the release of the Human1 model^19^. Here, as opposed to solely impairing cellular growth, a gene was defined as essential if it impaired any of 57 basic metabolic tasks, including functions such as de novo synthesis of ATP, oxidative phosphorylation and β-oxidation^19^. In this way, future model developments would benefit from an exploration of a mixed or alternative objective function, including other biological processes alongside cell growth, to determine how this would affect the sensitivity of flux analysis to gene expression constraints. Furthermore, this initial algorithm uses FBA, which outputs only a single, maximal objective solution, where many may be possible. Widely used algorithms, such as GIMME and E-Flux, utilise FBA and objective functions in their approach^22,24,25^, however, to refine our algorithm and consider a range of possible solutions and the probability of each of these solutions arising, future work should trial flux variability analysis and flux sampling. In addition, to apply an element of ‘confidence’ to prediction as to differential metabolism between OC subtypes, one could observe how robust the differential flux predictions are between multiple FBA iterations. In other words, out of a fixed number of FBA iterations, in how many solutions do we see the differential regulation of the same metabolic pathway? This is a point which could be considered for future projects employing FBA.

A potential future update to the methods described here would be the incorporation of proteomics data, to generate multi-omics models of OC metabolism. In this study, the integration of transcriptomics has been prioritised due to abundant transcriptomics databases, such as the CCLE and TCGA, thus maximising the accessibility of our algorithm to the cancer research community. Although not perfect, the CCLE reported a Pearson correlation between gene and protein expression of *r* = 0.48 across 375 cancer cell lines, which suggests some predictive potential^31^. Furthermore, transcriptomics has been used for this project due to its high-throughput nature^55^ and the fact that it provides an increased model coverage relative to proteomics, where CCLE datasets from 2020 included 9000 proteins compared to roughly 19,000 genes: approximately double the amount of molecules. We have optimised the predictive potential of transcriptomics in this algorithm by relaxing our reaction bounds to satisfy experimental growth rates, thereby deducing areas of gene expression which may not accurately reflect the proteome and growth phenotype. Furthermore, a multi-omics approach in ovarian research has allowed an integrative analysis of signalling pathways, and aided precision medicine spanning from genetic drivers to their downstream protein effectors; this provides an incentive for future multi-omics algorithm developments^56^.

Here we have used six cell line-specific models, for which the defined media was standardised to ensure the models were reflecting gene expression (rather than media-related) metabolic differences. The composition of FBS could only be estimated, using the reaction closing and reopening which has been described, due to a lack of published definition of this media. Therefore, future models would have their accuracy and reliability improved by incorporating a full chemical characterisation of FBS.

In conclusion, we have developed an ‘omics integration method for CBM, using the Human1 GEM framework and OC gene expression data. This work has shown that the use of experimental growth thresholds, and the relaxing of reaction bounds, can compensate for inconsistencies between gene expression and growth phenotype—a method which we have shown to have high efficacy in quickly proliferating cell lines. Various subtype-specific metabolic predictions have been made, including: the upregulation of the lactate shuttle and PPP in low-grade models, which supports fatty acid metabolism and redox balance, as well as potentiating chemoresistance; in high-grade models, the upregulation of fatty acid biosynthesis has been observed, which favours increased proliferation and a high energy demand of cells via the synthesis of long-chain fatty acids. Importantly, specific enzymes and molecules which showed subtype-specificity have been linked to existing targeted therapies, and their dysregulation is concordant with literature evidence. On top of this, experimental growth dependency scores have been able to validate the predicted low-grade-specific essentiality of the pentose phosphate pathway, with in silico KO simulations demonstrating the accuracy of CBM to predict gene essentiality and identify targets for growth inhibition. In this way, this work contributes to a greater understanding of the metabolic heterogeneity of OC, and the specific features responsible for contrasting chemosensitivities and survival prognosis observed for low- and high-grade subtypes. This method can be translated to other diseases and datasets, and these cell line-specific models provide a framework for future genetic engineering experiments and drug simulations, inspiring personalised medicine studies and improving the outlook for OC patients.

### Supplementary information

Supplementary Information

Supplementary Data 1

Supplementary Data 2

Supplementary Data 3

## Data Availability

Analysis results are available in Supplementary Data 1 to 3. The input dataset was obtained from the CCLE DepMap portal at https://depmap.org/portal/ccle/.

## Code availability

The code supporting this study is available at https://github.com/katemeeson/repository_to_accompany_paper_2023.

## References

[1] Momenimovahed, Z., Tiznobaik, A., Taheri, S. & Salehiniya, H. Ovarian cancer in the world: epidemiology and risk factors. *Int. J. Women’s Health* **11**, 287–299 (2019). 31118829 10.2147/IJWH.S197604 PMC6500433

[2] Reid, B. M., Permuth, J. B. & Sellers, T. A. Epidemiology of ovarian cancer: a review. *Cancer Biol. Med.* **14**, 9–32 (2017). 28443200 10.20892/j.issn.2095-3941.2016.0084 PMC5365187

[3] Jayson, G. C., Kohn, E. C., Kitchener, H. C. & Ledermann, J. A. Ovarian cancer. *Lancet Lond. Engl.* **384**, 1376–1388 (2014). 10.1016/S0140-6736(13)62146-7 24767708

[4] Shih, I.-M. & Kurman, R. J. Ovarian tumorigenesis. *Am. J. Pathol.* **164**, 1511–1518 (2004). 15111296 10.1016/S0002-9440(10)63708-X PMC1615664

[5] Cortez, A. J., Tudrej, P., Kujawa, K. A. & Lisowska, K. M. Advances in ovarian cancer therapy. *Cancer Chemother. Pharmacol.* **81**, 17–38 (2018). 29249039 10.1007/s00280-017-3501-8 PMC5754410

[6] Vang, R., Shih, Ie. M. & Kurman, R. J. Ovarian low-grade and high-grade serous carcinoma: pathogenesis, clinicopathologic and molecular biologic features, and diagnostic problems. *Adv. Anat. Pathol.* **16**, 267–282 (2009). 19700937 10.1097/PAP.0b013e3181b4fffa PMC2745605

[7] Warburg, O., Wind, F. & Negelein, E. The metabolism of tumors in the body. *J. Gen. Physiol.* **8**, 519–530 (1927). 19872213 10.1085/jgp.8.6.519 PMC2140820

[8] Nantasupha, C., Thonusin, C., Charoenkwan, K., Chattipakorn, S. & Chattipakorn, N. Metabolic reprogramming in epithelial ovarian cancer. *Am. J. Transl. Res* **13**, 9950–9973 (2021). 34650675 PMC8507042

[9] Ji, Z. et al. Deregulation of lipid metabolism: the critical factors in ovarian cancer. *Front. Oncol.* **10**, 593017 (2020). 33194756 10.3389/fonc.2020.593017 PMC7604390

[10] Ebrahim, A., Lerman, J. A., Palsson, B. O. & Hyduke, D. R. COBRApy: COnstraints-based reconstruction and analysis for python. *BMC Syst. Biol.* **7**, 74 (2013). 23927696 10.1186/1752-0509-7-74 PMC3751080

[11] Yizhak, K., Chaneton, B., Gottlieb, E. & Ruppin, E. Modeling cancer metabolism on a genome scale. *Mol. Syst. Biol.* **11**, 817 (2015). 26130389 10.15252/msb.20145307 PMC4501850

[12] Duarte, N. C. et al. Global reconstruction of the human metabolic network based on genomic and bibliomic data. *Proc. Natl. Acad. Sci. USA* **104**, 1777–1782 (2007). 17267599 10.1073/pnas.0610772104 PMC1794290

[13] Thiele, I. et al. A community-driven global reconstruction of human metabolism. *Nat. Biotechnol*. 10.1038/nbt.2488 (2013). 10.1038/nbt.2488 PMC3856361 23455439

[14] Brunk, E. et al. Recon3D enables a three-dimensional view of gene variation in human metabolism. *Nat. Biotechnol.* **36**, 272–281 (2018). 29457794 10.1038/nbt.4072 PMC5840010

[15] Mardinoglu, A. et al. Integration of clinical data with a genome-scale metabolic model of the human adipocyte. *Mol. Syst. Biol.* **9**, 649 (2013). 23511207 10.1038/msb.2013.5 PMC3619940

[16] Mardinoglu, A. et al. Genome-scale metabolic modelling of hepatocytes reveals serine deficiency in patients with non-alcoholic fatty liver disease. *Nat. Commun.* **5**, 3083 (2014). 24419221 10.1038/ncomms4083

[17] Thiele, I. et al. Personalized whole-body models integrate metabolism, physiology, and the gut microbiome. *Mol. Syst. Biol.* **16**, e8982 (2020). 32463598 10.15252/msb.20198982 PMC7285886

[18] Agren, R. et al. Identification of anticancer drugs for hepatocellular carcinoma through personalized genome-scale metabolic modeling. *Mol. Syst. Biol.* **10**, 721 (2014). 24646661 10.1002/msb.145122 PMC4017677

[19] Robinson, J. L. et al. An atlas of human metabolism. *Sci. Signal.* **13**, eaaz1482 (2020). 32209698 10.1126/scisignal.aaz1482 PMC7331181

[20] Motamedian, E., Ghavami, G. & Sardari, S. Investigation on metabolism of cisplatin resistant ovarian cancer using a genome scale metabolic model and microarray data. *Iran. J. Basic Med. Sci.* **18**, 267–276 (2015). 25945240 PMC4414993

[21] Arora, G., Banerjee, M., Langthasa, J., Bhat, R. & Chatterjee, S. Targeting metabolic fluxes reverts metastatic transitions in ovarian cancer. *iScience* **26**, 108081 (2023). 37876796 10.1016/j.isci.2023.108081 PMC10590820

[22] Blazier, A. S. & Papin, J. A. Integration of expression data in genome-scale metabolic network reconstructions. *Front. Physiol*. **3**, 299 (2012). 10.3389/fphys.2012.00299 PMC3429070 22934050

[23] Zur, H., Ruppin, E. & Shlomi, T. iMAT: an integrative metabolic analysis tool. *Bioinforma. Oxf. Engl.* **26**, 3140–3142 (2010). 10.1093/bioinformatics/btq602 21081510

[24] Becker, S. A. & Palsson, B. O. Context-specific metabolic networks are consistent with experiments. *PLoS Comput. Biol.* **4**, e1000082 (2008). 18483554 10.1371/journal.pcbi.1000082 PMC2366062

[25] Colijn, C. et al. Interpreting expression data with metabolic flux models: predicting Mycobacterium tuberculosis mycolic acid production. *PLoS Comput. Biol.* **5**, e1000489 (2009). 19714220 10.1371/journal.pcbi.1000489 PMC2726785

[26] Chandrasekaran, S. & Price, N. D. Probabilistic integrative modeling of genome-scale metabolic and regulatory networks in Escherichia coli and Mycobacterium tuberculosis. *Proc. Natl. Acad. Sci.* **107**, 17845–17850 (2010). 20876091 10.1073/pnas.1005139107 PMC2955152

[27] Lewis, J. E., Forshaw, T. E., Boothman, D. A., Furdui, C. M. & Kemp, M. L. Personalized genome-scale metabolic models identify targets of redox metabolism in radiation-resistant tumors. *Cell Syst.* **12**, 68–81.e11 (2021). 33476554 10.1016/j.cels.2020.12.001 PMC7905848

[28] Machado, D. & Herrgård, M. Systematic evaluation of methods for integration of transcriptomic data into constraint-based models of metabolism. *PLoS Comput. Biol.* **10**, e1003580 (2014). 24762745 10.1371/journal.pcbi.1003580 PMC3998872

[29] Ghandi, M. et al. Next-generation characterization of the Cancer Cell Line Encyclopedia. *Nature* **569**, 503–508 (2019). 31068700 10.1038/s41586-019-1186-3 PMC6697103

[30] Vieira, V., Ferreira, J. & Rocha, M. A pipeline for the reconstruction and evaluation of context-specific human metabolic models at a large-scale. *PLOS Comput. Biol.* **18**, e1009294 (2022). 35749559 10.1371/journal.pcbi.1009294 PMC9278738

[31] Nusinow, D. P. et al. Quantitative proteomics of the Cancer Cell Line Encyclopedia. *Cell* **180**, 387–402.e16 (2020). 31978347 10.1016/j.cell.2019.12.023 PMC7339254

[32] Barnes, B. M. et al. Distinct transcriptional programs stratify ovarian cancer cell lines into the five major histological subtypes. *Genome Med.* **13**, 140 (2021). 34470661 10.1186/s13073-021-00952-5 PMC8408985

[33] Lheureux, S., Gourley, C., Vergote, I. & Oza, A. M. Epithelial ovarian cancer. *Lancet* **393**, 1240–1253 (2019). 30910306 10.1016/S0140-6736(18)32552-2

[34] Domcke, S., Sinha, R., Levine, D. A., Sander, C. & Schultz, N. Evaluating cell lines as tumour models by comparison of genomic profiles. *Nat. Commun.* **4**, 2126 (2013). 23839242 10.1038/ncomms3126 PMC3715866

[35] Barretina, J. et al. The Cancer Cell Line Encyclopedia enables predictive modeling of anticancer drug sensitivity. *Nature* **483**, 603–607 (2012). 22460905 10.1038/nature11003 PMC3320027

[36] Wang, H. et al. SysBioChalmers/Human-GEM: Human 1.19.0. 10.5281/zenodo.4099692 (2022).

[37] Pereira, V., Cruz, F. & Rocha, M. MEWpy: a computational strain optimization workbench in Python. *Bioinformatics* **37**, 2494–2496 (2021). 33459757 10.1093/bioinformatics/btab013 PMC8388025

[38] Van Rossum, G. & Drake, F. L. The python language reference—Python 3.11.0 documentation. https://docs.python.org/3/reference/ (2009).

[39] Liao, Y., Wang, J., Jaehnig, E. J., Shi, Z. & Zhang, B. WebGestalt 2019: gene set analysis toolkit with revamped UIs and APIs. *Nucleic Acids Res.* **47**, W199–W205 (2019). 31114916 10.1093/nar/gkz401 PMC6602449

[40] Wagner, B. A. et al. Inactivation of anthracyclines by serum heme proteins. *Chem. Res. Toxicol.* **20**, 920–926 (2007). 17497896 10.1021/tx700002f PMC3617216

[41] Jerby, L. et al. Metabolic associations of reduced proliferation and oxidative stress in advanced breast cancer. *Cancer Res.* **72**, 5712–5720 (2012). 22986741 10.1158/0008-5472.CAN-12-2215

[42] Kanehisa, M. & Goto, S. KEGG: kyoto encyclopedia of genes and genomes. *Nucleic Acids Res.* **28**, 27–30 (2000). 10592173 10.1093/nar/28.1.27 PMC102409

[43] Kanehisa, M., Furumichi, M., Sato, Y., Kawashima, M. & Ishiguro-Watanabe, M. KEGG for taxonomy-based analysis of pathways and genomes. *Nucleic Acids Res*. 10.1093/nar/gkac963 (2022). 10.1093/nar/gkac963 PMC9825424 36300620

[44] Kanehisa, M. Toward understanding the origin and evolution of cellular organisms. *Protein Sci.* **28**, 1947–1951 (2019). 31441146 10.1002/pro.3715 PMC6798127

[45] McClelland, G. B., Khanna, S., González, G. F., Butz, C. E. & Brooks, G. A. Peroxisomal membrane monocarboxylate transporters: evidence for a redox shuttle system? *Biochem. Biophys. Res. Commun.* **304**, 130–135 (2003). 12705896 10.1016/S0006-291X(03)00550-3

[46] Katz, J. & Rognstad, R. Futile cycling in glucose metabolism. *Trends Biochem. Sci.* **3**, 171–174 (1978). 10.1016/S0968-0004(78)90980-5

[47] Li, X. et al. Lactate metabolism in human health and disease. *Signal Transduct. Target. Ther.* **7**, 305 (2022). 36050306 10.1038/s41392-022-01151-3 PMC9434547

[48] Yoon, H. & Lee, S. Fatty acid metabolism in ovarian cancer: therapeutic implications. *Int. J. Mol. Sci.* **23**, 2170 (2022). 35216285 10.3390/ijms23042170 PMC8874779

[49] Wang, M., Zhang, J. & Wu, Y. Tumor metabolism rewiring in epithelial ovarian cancer. *J. Ovarian Res.* **16**, 108 (2023). 37277821 10.1186/s13048-023-01196-0 PMC10240809

[50] Kobayashi, H., Imanaka, S. & Shigetomi, H. Revisiting therapeutic strategies for ovarian cancer by focusing on redox homeostasis. *Oncol. Lett*. **23**, 80 (2022). 10.3892/ol.2022.13200 PMC8771630 35111249

[51] Stincone, A. et al. The return of metabolism: biochemistry and physiology of the pentose phosphate pathway. *Biol. Rev.* **90**, 927–963 (2015). 25243985 10.1111/brv.12140 PMC4470864

[52] De Preter, G. et al. Inhibition of the pentose phosphate pathway by dichloroacetate unravels a missing link between aerobic glycolysis and cancer cell proliferation. *Oncotarget* **7**, 2910–2920 (2015). 10.18632/oncotarget.6272 PMC4823080 26543237

[53] Hidalgo, M. A., Carretta, M. D. & Burgos, R. A. Long chain fatty acids as modulators of immune cells function: contribution of FFA1 and FFA4 receptors. *Front. Physiol.* **12**, 668330 (2021). 34276398 10.3389/fphys.2021.668330 PMC8280355

[54] Chaudhry, S., Thomas, S. N. & Simmons, G. E. Jr. Targeting lipid metabolism in the treatment of ovarian cancer. *Oncotarget* **13**, 768–783 (2022). 35634242 10.18632/oncotarget.28241 PMC9132258

[55] Wang, Z., Gerstein, M. & Snyder, M. RNA-Seq: a revolutionary tool for transcriptomics. *Nat. Rev. Genet.* **10**, 57–63 (2009). 19015660 10.1038/nrg2484 PMC2949280

[56] Khella, C. A., Mehta, G. A., Mehta, R. N. & Gatza, M. L. Recent advances in integrative multi-omics research in breast and ovarian cancer. *J. Pers. Med.* **11**, 149 (2021). 33669749 10.3390/jpm11020149 PMC7922242

