# Supplementary material for: Constraint-based modelling predicts metabolic signatures of low and high-grade serous ovarian cancer
Source: NPJ Syst Biol Appl. 2024 Aug 24;10:96. doi: 10.1038/s41540-024-00418-5 (PMC11344801; doi:10.1038/s41540-024-00418-5)
Supplement: Supplementary file 1 — Supplementary Information [file 41540_2024_418_MOESM1_ESM.pdf]

Supplementary data

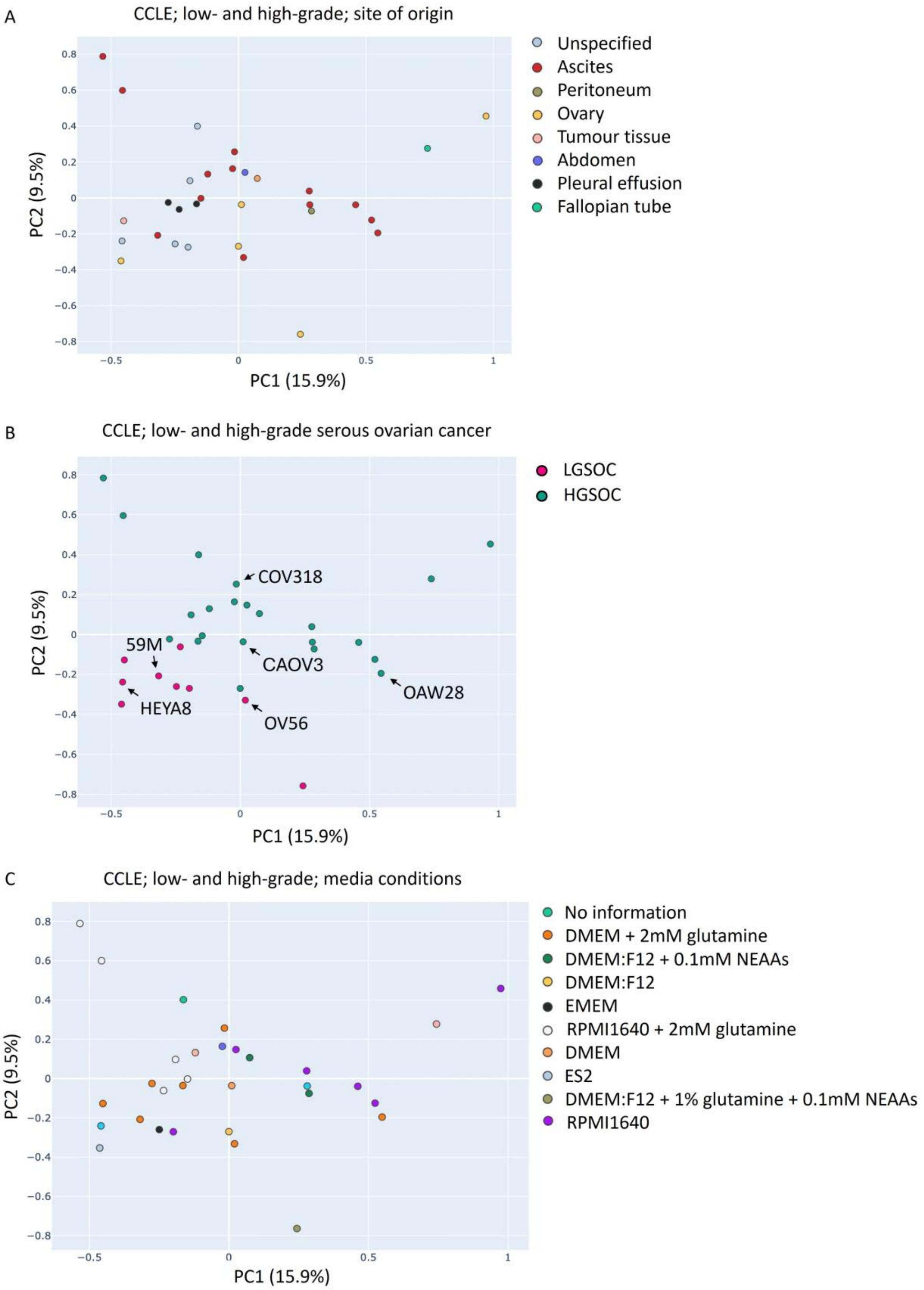

Supplementary Figure 1. Visualising how media and site of origin control spread of transcriptomics. Cancer cell line encyclopedia dataset, *n*=31 ovarian cell lines, low-grade and high-grade serous only. A. Cell line are coloured according to

site of origin. **B.** Cell line are coloured according to subtype (either low-grade or high-grade serous). Cell lines for which metabolic models have been constrained in this study have been indicated (COV318, CAOV3, OAW28, 59M, OV56, HEYA8). **C.** Cell line are coloured according to media on which the sample was grown. Where this was not available from the CCLE database, this has been labelled as ‘no information’.

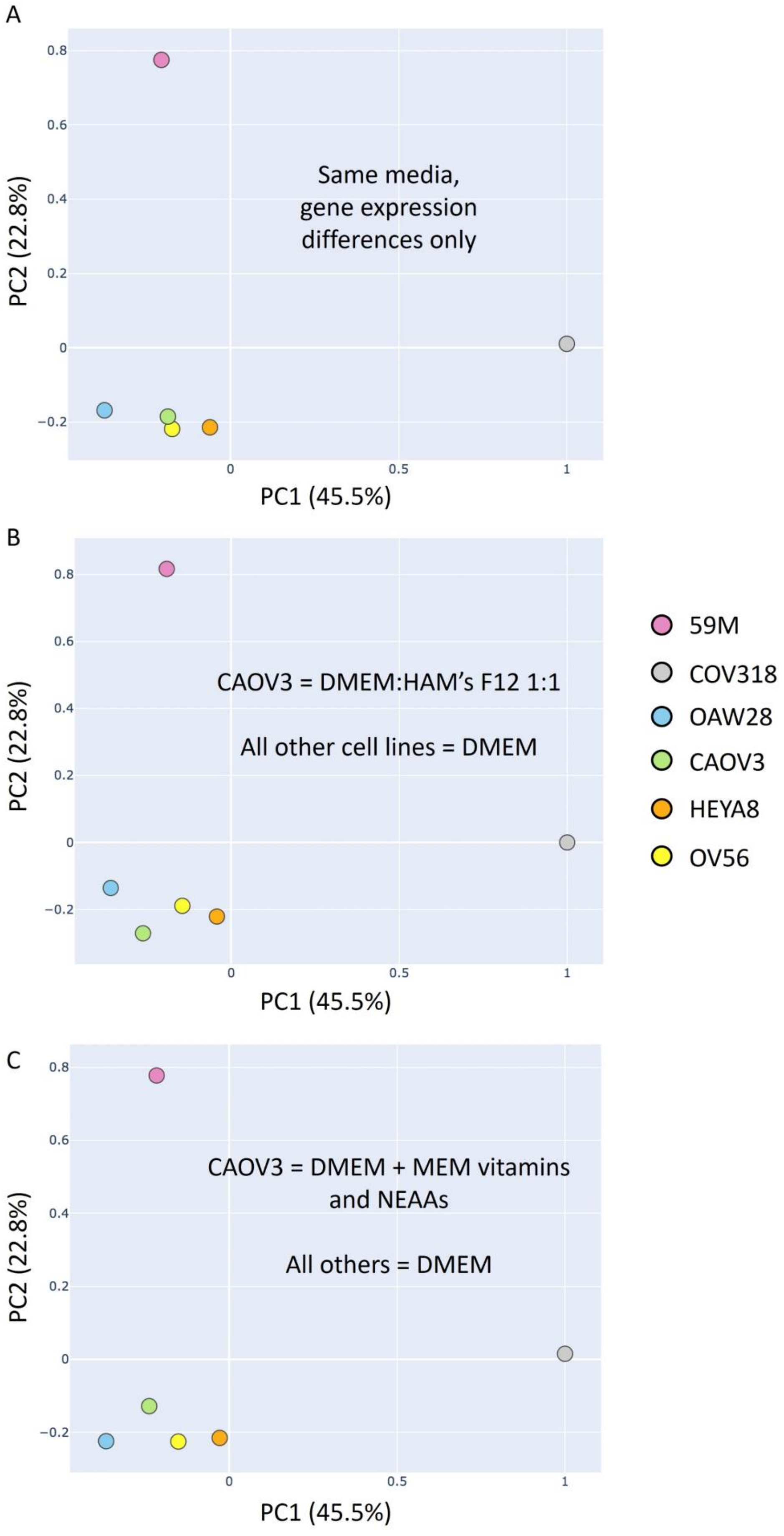
A
Same media,
gene expression
differences only
PC2 (22.8%)
PC1 (45.5%)
B
CAOV3 = DMEM:HAM's F12 1:1
All other cell lines = DMEM
PC2 (22.8%)
PC1 (45.5%)
59M
COV318
OAW28
CAOV3
HEYA8
OV56
C
CAOV3 = DMEM + MEM vitamins
and NEAAs
All others = DMEM
PC2 (22.8%)
PC1 (45.5%)

Supplementary Figure 2. Standardisation of media conditions. Cell line model indicated on key. Models constrained using Cancer Cell Line Encyclopedia gene expression data A. *In silico* media definition set to DMEM for all cell lines. **B.** *In silico* media definition set to DMEM for all cell lines, except CAOV3, where media definition was DMEM:HAM's F12 1:1 (as indicated on figure). **C.** *In silico* media definition set to DMEM for all cell lines, except CAOV3, where media definition was DMEM + MEM vitamins and non-essential amino acids (as indicated on figure).

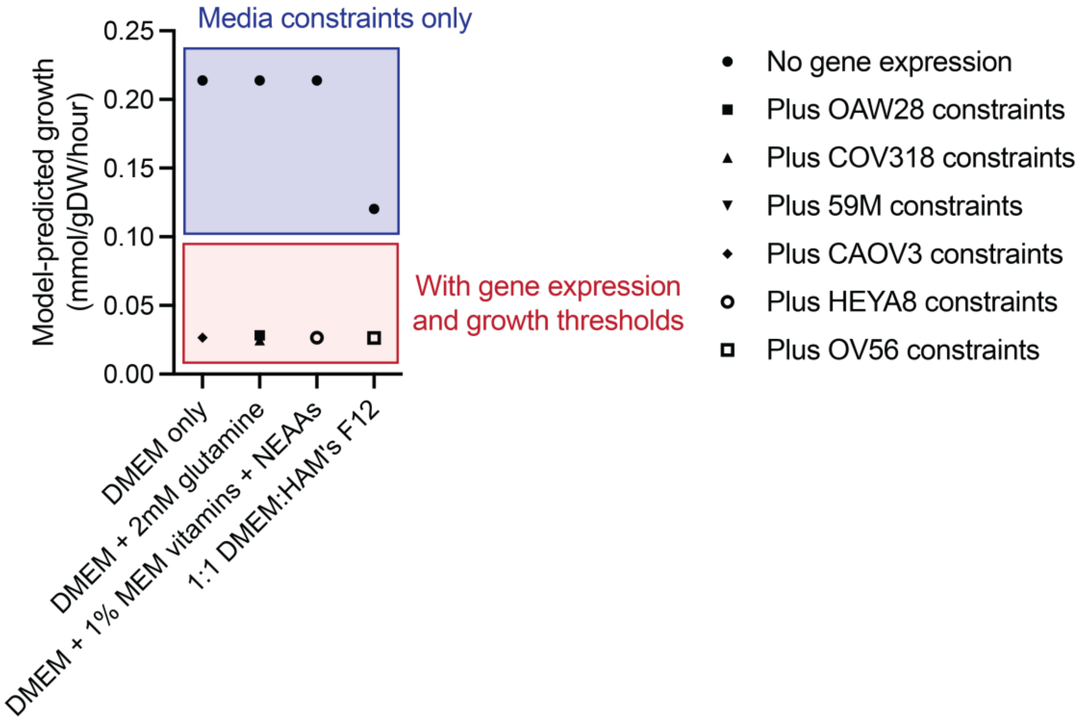

Supplementary Figure 3. Visualisation of the impact of 'omics constraints on FBA-predicted growth. Originally, models have been constrained only using media constraints, then their respective gene expression data (Cancer Cell Line Encyclopedia) was integrated into reaction bounds, and the new FBA-predicted growth rates have been plotted.

Supplementary Table 1. Media conditions for chosen CCLE cell lines

Cell line	Media composition	Reference for media composition
59M	DMEM + 2mM Glutamine + 1mM Sodium Pyruvate (NaP) + 20 IU/l Bovine Insulin + 10% Foetal Bovine Serum (FBS)	https://www.cellbankaustralia.com/59m.html
HEYA8	DMEM + 1% MEM vitamins + 1% MEM NEAAs + 10% FBS	PMID: 27147568
OV56	DMEM:HAMS F12 (1:1) + 2mM Glutamine + 5% Foetal Bovine Serum (FBS) + 0.5 ug/ml hydrocortisone + 10ug/ml insulin	https://www.culturecollections.org.uk/
CAOV3	DMEM + 10% FBS	https://www.atcc.org/products/htb-75
COV318	DMEM + 2mM Glutamine + 10% Foetal Bovine	https://www.culturecollections.org.uk/

	Serum (FBS)	
OAW28	DMEM + 2mM Glutamine + 1mM Sodium Pyruvate (NaP) + 20 IU/l Bovine Insulin + 10% Foetal Bovine Serum (FBS)	https://www.culturecollections.org.uk/

Supplementary Table 2. Experimental and model-predicted growth rates.

Cell line	Experimentally-predicted doubling time (hours)/growth rate (g/gDW/hour)	Source of experimental growth rate	Model-predicted doubling time (hours)/growth rate (g/gDW/hour)
59M	48/0.02083	Cellosaurus.org	37.66 (with growth threshold)<br>37.66 (with no growth threshold)
HEYA8	16/0.0625	Cellosaurus.org	15.79 (with growth threshold)<br>37.70 (with no growth threshold)
OV56	24.46/0.0409	PMID: 29774110	24.10 (with growth threshold)<br>38.02 (with no growth thresholds)
CAOV3	63.95/0.01564	Cellosaurus.org	38.70 (with growth threshold)<br>38.70 (with no growth threshold)
COV318	50/0.02	Cellosaurus.org	40.58 (with growth threshold)<br>40.58 (with no growth thresholds
OAW28	37/0.02703	Cellosaurus.org	35.47 (with growth threshold)<br>35.47 (with no growth threshold)

Supplementary files

Supplementary Data 2: Supplementary modeling worksheet

Supplementary Data 3: Knockout simulations

Supplementary Data 4: Media and subtypes

GitHub repository: https://github.com/katemeeson/repository_to_accompany_paper_2023

Bibliography

Barretina, J., Caponigro, G., Stransky, N., Venkatesan, K., Margolin, A. A., Kim, S., Wilson, C. J., Lehár, J., Kryukov, G. V., Sonkin, D., Reddy, A., Liu, M., Murray, L., Berger, M. F., Monahan, J. E., Morais, P., Meltzer, J., Korejwa, A., Jané-Valbuena, J., … Garraway, L. A. (2012). The Cancer Cell Line Encyclopedia enables predictive modeling of anticancer drug sensitivity. *Nature*, *483*(7391), 603–607. https://doi.org/10.1038/nature11003

Haley, J., Tomar, S., Pulliam, N., Xiong, S., Perkins, S. M., Karpf, A. R., Mitra, S., Nephew, K. P., & Mitra, A. K. (2016). Functional characterization of a panel of high-grade serous ovarian cancer cell lines as representative experimental models of the disease. *Oncotarget*, *7*(22), 32810–32820. https://doi.org/10.18632/oncotarget.9053

Tsherniak, A., Vazquez, F., Montgomery, P. G., Weir, B. A., Kryukov, G., Cowley, G. S., Gill, S., Harrington, W. F., Pantel, S., Krill-Burger, J. M., Meyers, R. M., Ali, L., Goodale, A., Lee, Y., Jiang, G., Hsiao, J., Gerath, W. F. J., Howell, S., Merkel, E., … Hahn, W. C. (2017). Defining a Cancer Dependency Map. *Cell*, *170*(3), 564-576.e16. https://doi.org/10.1016/j.cell.2017.06.010

Wilson, A. (2022a, November 14). *59M*. https://www.cellbankaustralia.com/59m.html

Wilson, A. (2022b, November 14). *ECACC General Cell Collection: OAW28*. Culture Collections. https://www.culturecollections.org.uk/products/celllines/generalcell/detail.jsp?refId=85101601&collection=ecacc_gc
